# Supplementary material for: Empirical methods for controlling false positives and estimating confidence in ChIP-Seq peaks
Source: BMC Bioinformatics. 2008 Dec 5;9:523. doi: 10.1186/1471-2105-9-523 (PMC2628906; doi:10.1186/1471-2105-9-523)
Supplement: Additional File 1 — A variety of html documents from the USeq web site detailing the available applications, their best usage, output file type descriptions, command line menus, etc. [file 1471-2105-9-523-S1.zip › USeqUserGuides/JavaDoc.zip!/JavaDoc/index-files/index-24.html]

Z-Index


|  |  |  |
| --- | --- | --- |
| **Overview** \| Package \| Class \| Use \| **Tree** \| **Deprecated** \| **Index** \| **Help** | |  |
| **PREV LETTER**   NEXT LETTER | **FRAMES**    **NO FRAMES**     **All Classes** |  |


A B C D E F G H I J L M N O P Q R S T U V W X Z 

---


## **Z**

**zeroedFloatArray(int, int)** - Static method in class util.gen.Num: Makes a float[x][y] and fills it with zeros. **zip(File, File)** - Static method in class util.gen.IO: Zip compress a single file. **zip(File[], File)** - Static method in class util.gen.IO: Zip compresses an array of Files, be sure to name your zipFile with a .zip extension! **ZipAndDelete** - Class in util.apps: **ZipAndDelete()** - Constructor for class util.apps.ZipAndDelete: **zipAndDelete(File)** - Static method in class util.gen.IO: Zip compresses the file and if sucessful, then deletes the original file. **zippedFile2StringArray(File)** - Static method in class util.gen.IO: Returns the contents of a zipped file. **zoom(int)** - Method in class gata.plotter.AlignPanel

---


|  |  |  |
| --- | --- | --- |
| **Overview** \| Package \| Class \| Use \| **Tree** \| **Deprecated** \| **Index** \| **Help** | |  |
| **PREV LETTER**   NEXT LETTER | **FRAMES**    **NO FRAMES**     **All Classes** |  |


A B C D E F G H I J L M N O P Q R S T U V W X Z 

---
